# Risk factor-targeted abdominal aortic aneurysm screening: systematic review of risk prediction for abdominal aortic aneurysm

**DOI:** 10.1093/bjs/znae239

**Published:** 2024-09-17

**Authors:** Liam Musto, Aiden Smith, Coral Pepper, Sylwia Bujkiewicz, Matthew Bown

**Affiliations:** Department of Cardiovascular Sciences, University of Leicester, NIHR Leicester Biomedical Research Centre, Glenfield Hospital, Leicester, UK; Department of Population Health Sciences, Biostatistics Research Group, University of Leicester, University Road, Leicester, UK; Library and Information Services, University Hospitals of Leicester NHS Trust, Leicester Royal Infirmary, Leicester, UK; Department of Population Health Sciences, Biostatistics Research Group, University of Leicester, University Road, Leicester, UK; Department of Cardiovascular Sciences, University of Leicester, NIHR Leicester Biomedical Research Centre, Glenfield Hospital, Leicester, UK

## Abstract

**Background:**

This systematic review aimed to investigate the current state of risk prediction for abdominal aortic aneurysm in the literature, identifying and comparing published models and describing their performance and applicability to a population-based targeted screening strategy.

**Methods:**

Electronic databases MEDLINE (via Ovid), Embase (via Ovid), MedRxiv, Web of Science, and the Cochrane Library were searched for papers reporting or validating risk prediction models for abdominal aortic aneurysm. Studies were included only if they were developed on a cohort or study group derived from the general population and used multiple variables with at least one modifiable risk factor. Risk of bias was assessed using the Prediction model Risk Of Bias ASsessment Tool. A synthesis and comparison of the identified models was undertaken.

**Results:**

The search identified 4813 articles. After full-text review, 37 prediction models were identified, of which 4 were unique predictive models that were reported in full. Applicability was poor when considering targeted screening strategies using electronic health record-based populations. Common risk factors used for the predictive models were explored across all 37 models; the most common risk factors in predictive models for abdominal aortic aneurysm were: age, sex, biometrics (such as height, weight, or BMI), smoking, hypertension, hypercholesterolaemia, and history of heart disease. Few models had undergone standardized model development, adequate external validation, or impact evaluation.

**Conclusion:**

This study identified four risk models that can be replicated and used to predict abdominal aortic aneurysm with acceptable levels of discrimination. None of the models have been validated externally.

## Teaser

This systematic review aimed to identify the current state and effectiveness of existing risk prediction models published for abdominal aortic aneurysm. The review identified 4 unique predictive models reported in full with acceptable levels of discrimination, and 33 additional predictive models not reported in full. None of the models had been validated externally. Common risk factors used across all models were identified, and applicability to targeted screening using electronic health records explored.

## Introduction

Abdominal aortic aneurysm (AAA) is a significant cause of mortality and morbidity. In England and Wales each year, AAA rupture causes over 3000 deaths and more than 7000 patients undergo surgical AAA repair to prevent rupture^1,2^.

Risk prediction models can be used to calculate the probability that an individual has a certain pathology or disorder; prognostic models work similarly to predict the probability or risk of future occurrence of a particular pathology or outcome. In healthcare, these models often use information about a patient’s demographics and associated co-morbidities to calculate the person’s risk/prognosis^3^.

Multiple trials have confirmed the long-term effectiveness of screening in reducing the risk of dying from ruptured AAA^4,5^. In the UK, the NHS AAA screening programme has been established since 2009, offering men in their 65th year a one-off ultrasound examination to screen for AAA^6^. Currently the programme detects an aneurysm in less than 1% of the men screened, and this number is expected to drop further in the future^7,8^. As disease prevalence falls over time, screening will become less and less cost-effective. Targeted screening has the potential to improve the cost-effectiveness of existing population-based AAA screening programmes. Any targeted screening strategy is likely to be based, at least in part, on AAA risk factors. In the USA, a targeted screening strategy for AAA has been established since 2005^9^. In this programme, men aged 65–75 years who have a history of smoking are recommended for screening with ultrasound imaging.

Electronic healthcare records (EHRs), such as those that exist in primary care or hospitals, offer an opportunity to apply clinical prediction models for screening, based on multiple risk factors (provided that these risk factors are recorded/detected in such records).

The aim of this review was to investigate the current state of risk prediction for AAA in the literature, identifying and comparing published models, and describing their performance and applicability to targeted screening strategies using EHR-based populations.

## Methods

A systematic literature review was undertaken in accordance with the PRISMA statement^10,11^. Before conducting the review, a protocol was developed and registered with PROSPERO (CRD42023395635)^12^.

With the aid of a clinical librarian, the following databases were searched for all relevant studies published before the search date (13 February 2024): MEDLINE (via Ovid), Embase (via Ovid), MedRxiv, Web of Science, and the Cochrane Library. The search strategy combined search terms used for the pathology ‘abdominal aortic aneurysm’ with the type of intervention ‘risk prediction model’ and appropriate combinations of Boolean operators ‘AND’, ‘OR’, and ‘NOT’; the full search strategy is available in the *supplementary material*.

Studies were eligible and included if they complied with the following criteria: article includes a risk prediction model that predicts the presence of an AAA; the prediction model is reported in full (in order that it can be replicated); the model was developed on a cohort or study group derived from the general population; and the model used multiple variables with at least one modifiable risk factor. Conference abstracts, editorials, case reports, and commentaries were excluded.

The aim was to identify the currently available risk prediction models for prediction of AAA in the general population and to evaluate their diagnostic accuracy.

Extracted information was based on the Cochrane guidance for data extraction and critical appraisal for systematic reviews of prediction models (CHARMS checklist)^3^. Measures of discrimination (ability to distinguish between those who do and do not have the outcome of interest) and calibration (agreement between predicted and observed number of events) were also extracted and summarized.

In the event of multiple studies validating a particular model, a meta-analysis would be conducted.

The literature search was performed by two reviewers, with any discrepancies checked by the senior author, on the 13 January 2023. A repeat search was conducted on 13 February 2024 after the analysis and before submission of the article to ensure that no models had been missed in the interim. The Covidence ^TM^ platform (Covidence, Melbourne, Victoria, Australia) was used to keep track of the process. No contact was made with the authors of screened literature. The titles and abstracts of all articles generated by the search strategy were screened for applicability. If the abstract met the inclusion criteria, the full paper was reviewed and assessed to ensure that it met the criteria for inclusion. All discrepancies were reviewed by the same three researchers and consensus was met regarding the eligibility of the study. After full-text review, citations and references used by each included publication were screened using the ResearchRabbit platform (ResearchRabbit, Seattle, WA, USA)^13^.

Risk-of-bias assessment was conducted using the Prediction model Risk Of Bias Assessment Tool (PROBAST)^14^, which explicitly evaluates four domains: bias arising from participants and data sources; bias owing to the predictors (assessment and definition); bias due to the outcome definition; and bias owing to data analysis and model fitting. The risk of bias was summarized as low, high, or unclear for each domain, and the evaluation similarly repeated for the first three domains in regard to applicability, producing seven scores for each study^15^. An overall assessment of risk of bias and applicability was then made at the end of this process, and similarly scored as low, high, or unclear.

## Results

**Fig. 1:**
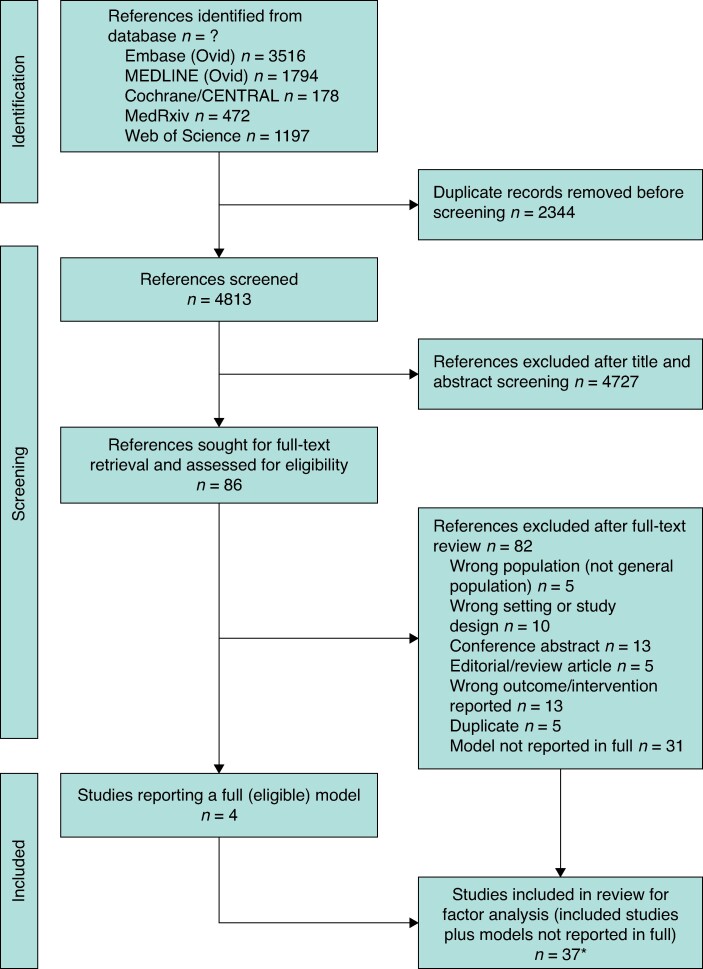
PRISMA diagram showing selection of articles for review *Citation and review of references was conducted on the included studies yielding no further included studies.

The search strategy yielded 4813 non-duplicate publications, which were then reviewed and screened manually (initially by title and abstract, then by full-text eligibility) by two reviewers, with discrepancies checked by the senior author^13^. Some 37 studies reported a multivariable model of some description, but only 4^16–19^ fulfilled the inclusion criteria fully; many papers failed to report the model in full or report a measure of performance. Reasons for exclusion and their frequency are outlined in *Fig. 1*. Details of all 37 studies are included in *Table S1*. Citation and reference review of included articles identified no further studies for inclusion from a total of 667 citations and references. The PRISMA flow chart is shown in *Fig. 1*.

Details of all the included studies and model performance are summarized in *Table 1*. Each model was developed in a different country of study^16^. Further details regarding the demographics of the derivation cohorts of these models are available in *Table S2*. All four models were developed using a logistic regression model and validation was performed using the development population, either by split-sample or *K*-fold cross-validation methods. Events per variable exceeded the widely advocated minimum criteria of 10 events per variable threshold for all 4 studies^20^. The level of discrimination of the models was excellent (over 0.8). None had been validated externally.

**Table 1:** Summary of models included in study

Reference Country	Handling of missing data	Predictor selection method	Validation method	No. of participants	No. of AAAs	EPV	Calibration	Discrimination	Variables included
**Kent *et al*.^19^** **USA**	Patients excluded	Backwards stepwise	Split sample (50 : 50)	3 056 455	23 446	1116	*R* ^2^ = 0.98, *P* < 0.001	C-statistic 0.842	Sex, age, ethnicity, hypertension, coronary artery disease, family history of AAA, high cholesterol, peripheral arterial disease, cerebrovascular disease, diabetes, history of CVD, smoking history, dietary factors, BMI
**Lanzarone *et al*.^18^** **Italy**	Patients excluded	Backwards stepwise	*K*-fold cross-validation	10 222	269	15	n.r.	C-statistic 0.838	Sex, age, cardiological status, BMI, diabetes, family history of CVD, pulmonary statis and dyspnoea, smoking status, hypertension, dyslipidaemia, chronic renal failure
**Pleumeekers *et al*.^17^** **Netherlands**	n.r.	Forward stepwise	n.r.	5283	112	16	n.r.	Sensitivity 0.94* Specificity 0.52*	Sex, age, smoking status, antihypertensive drug use, intermittent claudication
**Welsh *et al*.^16^** **UK**	Patients excluded	Forward stepwise	Split sample and bootstrapping (2000 reps)	400 250	1570	83	> 0.99†	C-statistic 0.889	Smoking status, age, weight, use of BP-lowering medication, statin use, sex, height, diabetes, diastolic BP, baseline previous CVD

*Multiple models provided; model 2 for risk of at least 1.5% selected. †At each of the 10 years of follow-up, the model calibration was above 0.99 in both the derivation and validation cohorts. AAA, abdominal aortic aneurysm; EPV, events per variable; CVD, cardiovascular disease; n.r., not reported.

PROBAST assessment of risk of bias and applicability is reported in *Table 2*. The risk of bias was considered high for all models. For example, despite the substantial cohort size, the study by Kent *et al.*^19^ was biased by including a highly selective cohort that reflected a healthier group; this was echoed by the low AAA prevalence in the cohort. All four models had poor applicability by either including predictors that might be lacking for an EHR-based targeted screening strategy (such as dietary behaviours or family history), or including an outcome that did not match those used by existing screening programmes (AAA threshold defined as 27 or 35 mm in the reported studies, rather than 30 mm).

**Table 2:** PROBAST assessment of bias

Reference	ROB				Applicability			Overall	
	Participants	Predictors	Outcome	Analysis	Participants	Predictors	Outcome	ROB	Applicability
**Kent *et al*.^19^**	–	+	+	+	+	–	+	–	–
**Lanzarone *et al*.^18^**	+	+	+	–	­–	+	–	–	–
**Pleumeekers *et al*.^17^**	–	+	+	–	–	+	–	–	–
**Welsh *et al*.^16^**	+	+	–	+	+	+	–	–	–

ROB, risk of bias; +, low risk; –, high risk.

Risk factors used in the 37 identified models (including the 4 fully reported models and those not fully reported that were predictive models for AAA, as well as those predicting aortic diameter as the primary outcome) are summarized in *Table 3*. The most common variables used were: smoking (29 of 37 models), modelled as history of smoking or ever smoked (16 of 29), pack-years smoked as a continuous variable (4 of 29), current smoking (11 of 29), and often including multiple smoking elements within the model; hypertension (25 of 37), modelled as a diagnostic history, those taking antihypertensive therapy, systolic and diastolic BP as continuous variables; age in years or categorized by decade (24 of 37); biometrics such as BMI, height, weight, body surface area or waist circumference (20 of 37); hypercholesterolaemia, reported as a diagnostic history, cholesterol level, diagnosis of dyslipidaemia, low-density lipoprotein cholesterol level, high-density lipoprotein cholesterol level or use of lipid-lowering therapy (20 of 37); sex (20 of 37); heart disease, modelled as a diagnostic history of cardiovascular disease, myocardial infarction, angina, coronary artery disease or coronary artery bypass, family history of cardiovascular disease or aspirin use (15 of 37).

**Table 3:** Factor analysis of 37 different models

Variable	Categories with notable variations listed in order of frequency (*n*)	No. of multivariable models using variable
**Smoking**	Ever smoked (16), current smoker (11), pack-years smoked (continuous variable) (4)	29
**Hypertension**	History of hypertension (19), on antihypertensive therapy (4), diastolic BP (4), systolic BP (3)	25
**Age**		24
**Body metrics**	BMI (11), height (6), weight (3), waist circumference (1), body surface area (2)	20
**Hypercholesterolaemia/dyslipidaemia**	Cholesterol (7), HDL-C (5), triglycerides (3), LDL-C (2), use of statin/lipid-lowering therapy (1), cholesterol : HDL ratio (1)	20
**Sex**		20
**Heart disease**	Coronary artery disease (6), history of myocardial infarction (5), history/presence of cardiovascular disease (4), family history of cardiovascular disease (2), angina (1), aspirin use (1), history of CABG (1)	15
**Diabetes**	Blood glucose level (1), HbA1c (2)	12
**Peripheral arterial disease**	Symptoms of intermittent claudication (1), ABPI (1)	8
**Cerebrovascular disease**	History of stroke/TIA (6), carotid stenosis (2), atrial fibrillation (1), CHADsVASC score (1)	7
**Ethnicity**		6
**Exercise level**	Daily walking/cycling extent (2), sedentary lifestyle (yes/no) (1)	6
**Family history of AAA**	Sister with AAA (1)	5
**Chronic renal failure**	Creatinine level (1), blood urea nitrogen level (1)	5
**Respiratory disease**	History of pulmonary disease (1), COPD (1), symptoms of dyspnoea characterized by NYHA score (1), FEV1 (1)	4
**Diet**	Consumption of nuts (2), fruit and vegetable consumption (1), anti-inflammatory diet (1), adherence to Mediterranean diet (1)	4
**Cancer/malignancy**	Myelogenous neoplasm (1)	2
**Complete blood count**	White blood cell count (2), red cell distribution width (1)	2
**Alcohol**	Alcohol consumption (1), presence of alcohol-related disease (1)	2
**Beta-blocker use**		1
**Place of birth**		1
**Other arterial aneurysm (personal history of)**		1

BP, blood pressure; BMI, body mass index; HDL-C, high-density lipoprotein cholesterol; LDL-C, low-density lipoprotein cholesterol; CABG, coronary artery bypass graft; HbA1c, haemoglobin A1c; ABPI, ankle brachial pressure index; TIA, transient ischaemic attack; AAA, abdominal aortic aneurysm; COPD, chronic obstructive pulmonary disease; NYHA, New York Heart Association; FEV1, forced expiratory volume in 1 s.

No model was investigated by more than one study and so no meta-analysis was conducted. Funnel plots were drawn to determine the presence or absence of publication bias; however, interpretability was limited by the small number of studies.

## Discussion

The intended purpose of the review was to determine all the AAA clinical prediction models available in the literature, and to identify models that could be used for a targeted screening programme. Most common variables were explored across the 37 models, and the commonest predictive factors for AAA were: smoking, hypertension, age, body metrics (such as height, weight or BMI), hypercholesterolaemia, sex/gender, and heart disease. The identified models demonstrated that it is possible to determine the risk of an individual having an AAA and to develop clinical algorithms to predict this. The validity of these models, however, was poorly assessed so their clinical value remains unknown. A predictive model using a combination of the factors identified during the present analysis is likely to be highly predictive of AAA, but this remains to be tested. A healthy lifestyle and smoking cessation, aiming to control some of these risk factors, may lower one’s risk of AAA.

The models identified came from studies carried out over different time intervals, with publication years ranging from 1997 to 2023. Environmental exposures, screening behaviours, aneurysm treatment as well as levels of statistical rigour have all changed considerably over that time. The four full models were published between 1999 and 2021. There is evidence that, even over that interval, the epidemiology of AAA changed considerably. AAA epidemiology has evolved so that AAA presents in older patients than previously and is less likely to rupture than in previous decades, leading to fewer deaths, emergency admissions, and repairs^8^. Thus, a key limitation of the applicability of these models is that any model developed before now may not be applicable to current patients with vascular disease as the dominant risk factors have evolved over time. Another limitation of the use of these models is the variation in diagnostic criteria for AAA. Most studies used ultrasound screening as the diagnostic modality, but only two of the four included studies used the fixed threshold diameter of 30 mm (1 of which was derived from ICD-10 coding indirectly). A strength of this study is the comprehensive search strategy that included major databases, reference review, and citation links. The study was limited by the fact that almost none of these models were developed specifically to be predictive models for AAA; many were designed to identify potent risk factors for AAA or to optimize screening programmes already in place. Direct comparison of the models was not possible owing to marked heterogeneity between studies, and no model was studied more than once to allow meta-analysis. Few studies had published model performance measures or performed validation methods, reflecting poor transparency in the vascular literature in regard to the development of prediction models. Few had undergone standardized model development, and none had been subjected to adequate external validation or impact evaluation.

The quality of the identified models was, therefore, surprisingly poor considering the methodological advances in the area of risk prediction modelling and existing literature providing guidelines for analysts^21–24^.

A future targeted screening programme is expected to use a complex predictive model such as those reported in this study. It is likely that it will be based on risk factors such as those identified in *Table 3*, using existing electronic resources such as EHRs rather than questionnaires. Thus, because of the limitations highlighted above and also the absence of well recorded data on certain risk factors in existing records, the four fully reported models identified would not be immediately applicable for use in a targeted screening programme using EHRs for the detection of AAA.

With the dawn of artificial intelligence and the era of big data, traditional development of predictive models is likely to be superseded by more complex machine learning techniques. This is to be welcomed, provided that the black box is kept open, methods are transparent, and biases explored rigorously. It is worth noting that, currently, machine learning is still less effective than standard statistical modelling in many fields, including prognostic research^25,26^. The future is likely to see clinical prediction models move to a more precision health method, with prediction based not on modifiable risk factors and presentations of disease but the genetics of the patient or identifiable biomarkers. Polygenic risk scores have demonstrated strong associations with AAA, and are even able to identify subgroups at distinctly higher risk who may benefit from alternative means of clinical management^27^. Combination of polygenic risk score and EHR data has already been proven to provide a model with strong association of AAA risk with discrimination (area under the curve 0.883) comparable to that of all the models identified here^28^. This is only expected to improve as genome databases grow, and computing power improves. However, the ethical concerns surrounding widespread use of genomic data for targeted population-based screening programmes cannot be overlooked.

## Supplementary Material

znae239_Supplementary_Data

## Data Availability

All relevant data are within the manuscript and its supporting information.

## Supplementary material

Supplementary  material is available at *BJS* online.
