# Supplementary material for: Risk factor-targeted abdominal aortic aneurysm screening: systematic review of risk prediction for abdominal aortic aneurysm
Source: Br J Surg. 2024 Sep 17;111(9):znae239. doi: 10.1093/bjs/znae239 (PMC11406543; doi:10.1093/bjs/znae239)
Supplement: znae239_Supplementary_Data [file znae239_supplementary_data.zip › Supplementary_Material_Not_for_Review_2.docx]

**Risk factor targeted AAA screening: A systematic review of risk prediction for AAA.**

Liam Musto^1^, Aiden Smith^3^, Coral Pepper^2^, Sylwia Bujkiewicz^3^, Matt Bown^1^

^1^Department of Cardiovascular Sciences, University of Leicester, NIHR Leicester Biomedical Research Centre, Glenfield Hospital, Leicester, LE3 9QP, United Kingdom

^2^Library and Information Services, University Hospitals of Leicester NHS Trust, Leicester Royal Infirmary, Infirmary Square, Leicester, LE1 5WW, United Kingdom

^3^Biostatistics Research Group, Department of Population Health Sciences, University of Leicester, University Road, Leicester, LE1 7RH, United Kingdom.

**Corresponding author.**

Liam Musto

Address: Vascular Research Office, Department of Cardiovascular Sciences, University of Leicester, NIHR Leicester Biomedical Research Centre, Glenfield Hospital, Leicester, LE3 9QP, United Kingdom.

Email: lm503@leicester.ac.uk, Telephone: 0116 2502381

**ORCID ID**; 0000-0002-7559-0063

**Twitter**; @Isntthtpleasant

**Supplementary Materials - Index**

| **Supplementary Methods** |  |
| --- | --- |
| Systematic review search strategy | *page 2 -5* |
| **Supplementary Figures and Tables** |  |
| Table S1 | *page 6* |
| Table S2 | *page 7* |

**Supplementary Methods: Systematic review search strategy**

**Ovid MEDLINE(R) ALL <1946 to January 10, 2023>** **(Run 11^th^ January 2023)**

1 (("abdom* aort*" or "aort* abdom*") adj3 aneurysm*).tw. 22189

2 "triple A".tw. 339

3 AAA.tw. 15604

4 Aortic Aneurysm, Abdominal/ 21910

5 or/1-4 35766

6 (risk* adj2 (model* or score* or equation* or calculat* or predict* or stratif*9 or function* or criteria or characteristic*)).tw,kw,kf. 175878

7 (predict* adj2 (model* or score* or equation* or calculat* or inciden* or function* or outcome* or factor* or criteria or characteristic*)).tw,kw,kf. 345442

8 (probabilit* adj2 (model* or score* or equation* or calculat* or function* or outcome* or factor* or criteria or characteristic*)).tw,kw,kf. 17301

9 (prognostic adj2 (model* or score* or equation* or calculat* or function* or outcome* or factor* or criteria or characteristic*)).tw,kw,kf. 144197

10 (decision* adj2 model*).tw,kw,kf. 13193

11 (statistic* adj2 model*).tw,kw,kf. 30555

12 "multivaria* model*".tw,kw,kf. 33617

13 (multicomponent adj2 model*).tw,kw,kf. 436

14 "logistic model*".tw,kw,kf. 10418

15 "hazard* model*".tw,kw,kf. 52027

16 (observ* adj2 (variation or model*)).tw,kw,kf. 22626

17 models, statistical/ 98665

18 protective factors/ 6140

19 exp Disease Susceptibility/cl, di, ep, et, pc [Classification, Diagnosis, Epidemiology, Etiology, Prevention & Control] 6490

20 or/6-19 837140

21 5 and 20 1628

**(additional line run 13^th^ February 2024):**

22 limit 21 to yr="2023 - 2024" 162

**Embase <1974 to 2023 January 10> (Run 11^th^ January 2023)**

1 (("abdom* aort*" or "aort* abdom*") adj3 aneurysm*).tw. 27932

2 "triple A".tw. 507

3 AAA.tw. 21738

4 exp abdominal aortic aneurysm/ 10395

5 or/1-4 41319

6 (risk* adj2 (model* or score* or equation* or calculat* or predict* or stratif*9 or function* or criteria or characteristic*)).tw,kw,kf. 280864

7 (predict* adj2 (model* or score* or equation* or calculat* or inciden* or function* or outcome* or factor* or criteria or characteristic*)).tw,kw,kf. 491095

8 (probabilit* adj2 (model* or score* or equation* or calculat* or function* or outcome* or factor* or criteria or characteristic*)).tw,kw,kf. 21699

9 (prognostic adj2 (model* or score* or equation* or calculat* or function* or outcome* or factor* or criteria or characteristic*)).tw,kw,kf. 218856

10 (decision* adj2 model*).tw,kw,kf. 18594

11 (statistic* adj2 model*).tw,kw,kf. 38007

12 "multivaria* model*".tw,kw,kf. 54767

13 (multicomponent adj2 model*).tw,kw,kf. 490

14 "logistic model*".tw,kw,kf. 14528

15 "hazard* model*".tw,kw,kf. 86003

16 (observ* adj2 (variation or model*)).tw,kw,kf. 28362

17 exp cardiovascular risk/ and (model* or score* or equation* or calculat* or stratif*9).tw. 72422

18 disease predisposition/ and (model* or score* or equation* or calculat* or stratif*9).tw. 21139

19 prediction/ and (model* or score* or equation* or calculat* or stratif*9).tw. 252026

20 exp statistical model/ 671032

21 or/6-20 1786463

22 5 and 21 3213

**(additional line run 13^th^ February 2024):**

23 limit 22 to yr="2023 - 2025" 303

**Cochrane Central Register of Controlled Trials (CENTRAL)**

**153 results on 11^th^ January 2023**

#1 (((abdom* NEXT aort*) or (aort* NEXT abdom*)) NEAR/3 aneurysm*) 1427

#2 "triple A" 247

#3 AAA 1548

#4 MeSH descriptor: [Aortic Aneurysm, Abdominal] this term only 618

#5 {OR #1-#4} 2674

#6 (risk* NEAR/2 (model* or score* or equation* or calculat* or predict* or stratif* or function* or criteria or characteristic*)) 20869

#7 (predict* NEAR/2 (model* or score* or equation* or calculat* or inciden* or function* or outcome* or factor* or criteria or characteristic*)) 22093

#8 (probabilit* NEAR/2 (model* or score* or equation* or calculat* or function* or outcome* or factor* or criteria or characteristic*)) 1749

#9 (prognostic NEAR/2 (model* or score* or equation* or calculat* or function* or outcome* or factor* or criteria or characteristic*)) 9624

#10 decision* NEAR/2 model* 1213

#11 statistic* NEAR/2 model* 7354

#12 multivaria* NEXT model* 3147

#13 multicomponent NEAR/2 model* 37

#14 logistic NEXT model* 5956

#15 hazard* NEXT model* 13035

#16 (observ* NEAR/2 (variation or model*)) 3287

#17 MeSH descriptor: [Models, Statistical] explode all trees 16387

#18 MeSH descriptor: [Protective Factors] this term only 144

#19 MeSH descriptor: [Disease Susceptibility] explode all trees and with qualifier(s): [classification - CL, diagnosis - DI, etiology - ET, epidemiology - EP] 127

#20 {OR #6-#19} 78682

#21 #5 AND #20 342 (153 in CENTRAL)

**(additional line run 13^th^ February 2024):**

#22 #21 with Cochrane Library publication date in The last year 23

**medrXiv – 11^th^ January 2023**

("abdominal aortic aneurysm" OR AAA) AND (predict* OR risk*) AND model* - 365 results

**(additional search run 13^th^ February 2024):**

"("abdominal aortic aneurysm" OR AAA) AND (predict* OR risk*) AND model* " and posted between "13 Jan, 2023 and 13 Feb, 2024" – 116 results

**WEB OF SCIENCE – 13^th^ January 2023**

(((AB=(abdom* NEAR/3 aneurysm* )) AND TI=(abdom* NEAR/3 aneurysm* )) AND TI=(aort* NEAR/3 aneurysm* )) AND AB=(aort* NEAR/3 aneurysm* )

1. abdom* NEAR/3 aneurysm* (title)

1 aort* NEAR/3 aneurysm* (title)

1 abdom* NEAR/3 aneurysm* (abstract)

1 aort* NEAR/3 aneurysm* (abstract) 8691

2 **(TI=(AAA)) AND AB=(AAA) 1512**

3 ALL=(abdominal aortic aneurysm) 28,597

4 or/1-3

6 (AB=(risk* NEAR/2 Model*)) OR AB=(risk* NEAR/2 score*) OR (TI=(risk* NEAR/2 Model*)) OR TI=(risk* NEAR/2 score*) 108,222

7 (AB=(predict* NEAR/2 Model*)) OR AB=(Predict* NEAR/2 score*) OR (TI=(predict* NEAR/2 Model*)) OR TI=(Predict* NEAR/2 score*) 553030

8 (AB=(probabilit* NEAR/2 model*)) OR AB=(probabilit* NEAR/2 score*) OR (TI=(probabilit* NEAR/2 model*)) OR TI=(probabilit* NEAR/2 score*) 29,403

9 (AB=(prognostic* NEAR/2 model*)) OR AB=(prognostic* NEAR/2 score*) OR (TI=(prognostic* NEAR/2 model*)) OR TI=(prognostic* NEAR/2 score*) 20,834

10 (TI=(decision* NEAR/2 model*)) OR AB=(decision* NEAR/2 Model*) 52116

11 **(**TI=(statistic* NEAR/2 model*)) OR AB=(statistic* NEAR/2 Model*) 108795

12 **(TI=(multivaria* NEAR/2 model*)) OR AB=(multivaria* NEAR/2 model*) 103,749**

13 **(TI=(multicomponent NEAR/2 model*)) OR AB=(multicomponent NEAR/2 model*) 3149**

14 (TI=(logistic model*)) OR AB=(logistic model*) 206,237

15 (TI=(hazard model*)) OR AB=(hazard model*) 146,916

16 or/6-15 1,162,932

21 4 and 16 1093

Added keyword plus

17 **KP=("AAA")**

18 **KP=("abdominal aortic aneurysm")**

**4 OR 17 OR 18 31,698**

**TOTAL 1099 papers**

**(additional search run 13^th^ February 2024):**

(publication date after 2023-01-13, TOTAL 98 papers)

**Supplementary Figures and Tables**

**Table S1:**

| **Table S1: Details of the 37 included studies** | | | |
| --- | --- | --- | --- |
| **Title** | **Year of Study** | **DOI Link** | **Included (Yes/No)** |
| Risk factors associated with increased prevalence of abdominal aortic aneurysm in women | 2016 | https://doi.org/10.1002/bjs.10179 | N |
| Prediction of the Probability and Risk Factors of Early Abdominal Aortic Aneurysm Using the Gradient Boosted Decision Trees Model | 2022 | https://doi.org/10.1080/08839514.2021.2014190 | N |
| Risk factors in 50-year-old men predicting development of abdominal aortic aneurysm. | 2020 | https://doi.org/10.1016/j.jvs.2019.11.062 | N |
| Analysis of risk factors for abdominal aortic aneurysm in a cohort of more than 3 million individuals | 2010 | https://doi.org/10.1016/j.jvs.2010.05.090 | Y |
| Prediction model of isolated iliac and abdominal aneurysms. | 2021 | https://doi.org/10.1111/eci.13517 | Y |
| Selecting subjects for ultrasonographic screening for aneurysms of the abdominal aorta: four different strategies. | 1999 | https://doi.org/10.1093/ije/28.4.682 | Y |
| Development and validation of risk prediction models for multiple cardiovascular diseases and Type 2 diabetes. | 2020 | https://doi.org/10.1371/journal.pone.0235758 | N |
| Abdominal aortic aneurysm screening program using hand-held ultrasound in primary healthcare | 2017 | https://doi.org/10.1371/journal.pone.0176877 | N |
| Derivation and Validation of a 10-Year Risk Score for Symptomatic Abdominal Aortic Aneurysm: Cohort Study of Nearly 500 000 Individuals. | 2021 | https://doi.org/10.1161/circulationaha.120.053022 | Y |
| Analysis of High-Risk Factors Associated with the Progression of Subaneurysmal Aorta to Abdominal Aortic Aneurysm in Rural Area in China. | 2021 | https://doi.org/10.2147/cia.s321921 | N |
| Selective Screening for Abdominal Aortic Aneurysm | 1997 | PMID: 9079351* | N |
| A Simple Risk Scoring Systems to evaluate the presence of aneurysm and one-year mortality in patients with abdominal aortic aneurysm using CHA2DS2-VASc and ATRIA | 2021 | https://doi.org/10.1590/1806-9282.67.01.20200487 | N |
| Anti-inflammatory diet and risk of abdominal aortic aneurysm in two Swedish cohorts | 2019 | https://dx.doi.org/10.1136/heartjnl-2019-315030 | N |
| Association between abdominal aortic aneurysms and alcohol-related diseases. | 2020 | https://dx.doi.org/10.1097/MD.0000000000022968 | N |
| Middle Age Cardiovascular Risk Factors and Abdominal Aortic Aneurysm in Older Age | 2003 | https://doi.org/10.1161/01.hyp.0000078829.02288.98 | N |
| Plasma ferritin concentrations are not associated with abdominal aortic aneurysm diagnosis, size or growth | 2016 | https://doi.org/10.1016/j.atherosclerosis.2016.05.022 | N |
| The epidemiology of abdominal aortic diameter | 2008 | https://doi.org/10.1016/j.jvs.2008.02.031 | N |
| Gender-specific Predicted Normal Aortic Size and Its Consequences of the Population-Based Prevalence of Abdominal Aortic Aneurysms | 2023 | https://doi.org/10.1016/j.avsg.2022.11.025 | N |
| Traditional and Novel Risk Factors for Clinically Diagnosed Abdominal Aortic Aneurysm: The Kaiser Multiphasic Health Checkup Cohort Study | 2007 | https://doi.org/10.1016/j.annepidem.2007.02.004 | N |
| Population risk factor estimates for abdominal aortic aneurysm from electronic medical records: a case control study | 2014 | https://doi.org/10.1186/1471-2261-14-174 | N |
| Obesity and abdominal aortic aneurysm | 2013 | https://doi.org/10.1002/bjs.8983 | N |
| Statistical and machine learning methodology for abdominal aortic aneurysm prediction from ultrasound screenings | 2019 | https://doi.org/10.1111/echo.14519 | N |
| Is the aortic size index relevant as a predictor of abdominal aortic aneurysm? A population-based prospective study: the Tromsø study | 2019 | https://doi.org/10.1080/14017431.2019.1707864 | N |
| Risk factors for abdominal aortic aneurysm in the Korean population | 2016 | https://doi.org/10.3390/jcm12020484 | N |
| Lifestyle and Risk of Screening-Detected Abdominal Aortic Aneurysm in Men | 2017 | https://doi.org/10.1161/jaha.116.004725 | N |
| Height and Mortality from Aortic Aneurysm and Dissection | 2022 | https://doi.org/10.5551%2Fjat.62941 | N |
| Smoking, sex, risk factors and abdominal aortic aneurysms: a prospective study of 18 782 persons aged above 65 years in the Southern Community Cohort Study | 2015 | https://doi.org/10.1136/jech-2014-204920 | N |
| Lifetime Risk and Risk Factors for Abdominal Aortic Aneurysm in a 24 Year Prospective Study: the ARIC Study | 2016 | https://doi.org/10.1161%2FATVBAHA.116.308147 | N |
| Chronic kidney disease measures and the risk of abdominal aortic aneurysm | 2019 | https://doi.org/10.1016%2Fj.atherosclerosis.2018.08.043 | N |
| Modifiable risk factor burden and the prevalence of peripheral artery disease in different vascular territories | 2013 | https://doi.org/10.1016/j.jvs.2013.01.053 | N |
| Thoracic and abdominal aortic diameters in a general population: MRI-based reference values and association with age and cardiovascular risk factors | 2016 | https://doi.org/10.1007/s00330-015-3926-6 | N |
| Development of a Novel Scoring Tool for the Identification of Large 5 cm Abdominal Aortic Aneurysms | 2010 | https://doi.org/10.1097/sla.0b013e3181f621c8 | N |
| The potential for a selective screening strategy for abdominal aortic aneurysm | 2000 | https://doi.org/10.1136/jms.7.4.209 | N |
| Prevalence of Peripheral Arterial Disease, Abdominal Aortic Aneurysm, and Risk Factors in the Hamburg City Health Study: A Cross Sectional Analysis | 2023 | https://doi.org/10.1016/j.ejvs.2023.01.002 | N |
| Association between the non-HDL-cholesterol to HDLcholesterol ratio and abdominal aortic aneurysm from a Chinese screening program | 2023 | https://doi.org/10.1186%2Fs12944-023-01939-4 | N |
| Prevalence and Risk Factors of Abdominal Aortic Aneurysms Detected with Ultrasound in Korea and Belgium | 2023 | https://doi.org/10.3390/jcm12020484 | N |
| *where no DOI link available a pubmed ID is provided | | | |

**Table S2:**

| **Table S2: Demographics and details of the derivation cohorts of the four included full models** | | | | | | |
| --- | --- | --- | --- | --- | --- | --- |
| **Paper Title and country of study** | **Year of Publication** | **Number of Participants** | **Gender Details** | **Age Details (mean), Years** | **Smoking behaviours** | **Definition of AAA** |
| **Kent et al. (USA)** | 2010 | 3056455 Total cohort  1528228 Derivation cohort | 65.1% Female*  34.9% Male* | 63.1* | 42.8% Smokers*  (80.22% of AAA cases*) | Aortic diameter on ultrasound >= 30mm |
| **Lanzarone et al. (Italy)** | 2021 | 10842 Total cohort | 11.9% Female  88.1% Male | Approximately** 70.06* | Not given | Aortic diameter on ultrasound >= 27mm |
| **Pleumeekers et al. (Netherlands)** | 1999 | 5283 Total cohort | 58% Female  42% Male | 67.7 | 3.1% History of smoking  23.5% current smoking | Aortic diameter on ultrasound >= 35mm |
| **Welsh et al. (UK)** | 2021 | 485636 Total cohort  401820 Derivation Cohort | 54.6% Female  45.4% Male | 56.4 | 45.0% History of smoking  (79.2% of AAA cases) | Hospital record coding ICD-10 codes I71.3 and I71.4 |
| *Given for the entire cohort  **calculated approximately by eliminating less than and greater than signs from totals table given in paper | | | | | | |
